# Parenteral systems for statin delivery: a review

**DOI:** 10.1186/s12944-019-1139-8

**Published:** 2019-11-05

**Authors:** Shahla Korani, Samira Bahrami, Mitra Korani, Maciej Banach, Thomas P. Johnston, Amirhossein Sahebkar

**Affiliations:** 1 Research center of oils and fats, Kermanshah University of Medical Sciences, Kermanshah, Iran; 2 Biotechnology Department, School of Advanced Technologies in Medicine, Shahid Beheshti University of Medical Sciences, Tehran, Iran; 3 Nanotechnology Research Center, Buali (Avicenna) Research Center, Mashhad University of Medical Sciences, Mashhad, Iran; 4 Department of Hypertension, WAM University Hospital in Lodz, Medical University of Lodz, Zeromskiego 113, Lodz, Poland; 5 Polish Mother’s Memorial Hospital Research Institute (PMMHRI), Lodz, Poland; 6 Division of Pharmacology and Pharmaceutical Sciences, School of Pharmacy, University of Missouri-Kansas City, Kansas City, Missouri USA; 7 Neurogenic Inflammation Research Center, Mashhad University of Medical Sciences, Mashhad, Iran; 8 Biotechnology Research Center, Pharmaceutical Technology Institute, Mashhad University of Medical Sciences, Mashhad, 9177948564 Iran; 9 School of Pharmacy, Mashhad University of Medical Sciences, Mashhad, Iran; 10 Department of Medical Biotechnology, School of Medicine, Mashhad University of Medical Sciences, P.O. Box: 91779-48564, Mashhad, Iran

**Keywords:** Statins, Cholesterol, Parenteral, Drug delivery

## Abstract

The oral route of drug administration is the most common and convenient route for dosing statin drugs, and, in fact, most medications, because of ease of drug delivery, patient compliance, and cost-effectiveness. However, the oral administration of statin drugs has disadvantages such as hepatic first-pass metabolism and degradation within the gastrointestinal tract that limit their overall bioavailability. This review introduces several diverse non-oral delivery methods for the administration of statins. These alternative delivery systems and routes of administration are varied and are capable of improving the bioavailability and therapeutic efficacy of statin drugs.

## Introduction

Currently, statin drugs are generally administered to patients by the oral route of drug administration. In fact, this is the only FDA-approved route of drug administration for statins. Administration of drugs by the oral route remains the most convenient and common method by which drugs are administered to human patients. Some of the reasons why the oral route of drug delivery remains popular is because of ease of administration, greater patient compliance, sterility requirements that are less stringent than sterile parenteral products, and lower cost both for the producer and consumer. However, there are some limitations associated with the oral administration of statins. For instance, the bioavailability of statin drugs is rather low due to both metabolism in the gut wall and subsequent ‘first-pass’ metabolism in the liver [1]. Moreover, there are additional factors that limit the oral bioavailability of statins, including drug permeability, suboptimal water solubility, drug efflux pathways, and direct, efficient transport to hepatocytes and subsequent binding to receptors on the rate-limiting enzyme for cholesterol biosynthesis; namely, 3-hydroxy-3-methyglutaryl Coenzyme A (HMG-CoA) reductase [2]. Taken together, pharmaceutical scientists have investigated alternative routes of drug administration, as well as a variety of pharmaceutical formulations, for statin drugs in order to enhance their bioavailability.

**Fig. 1:**
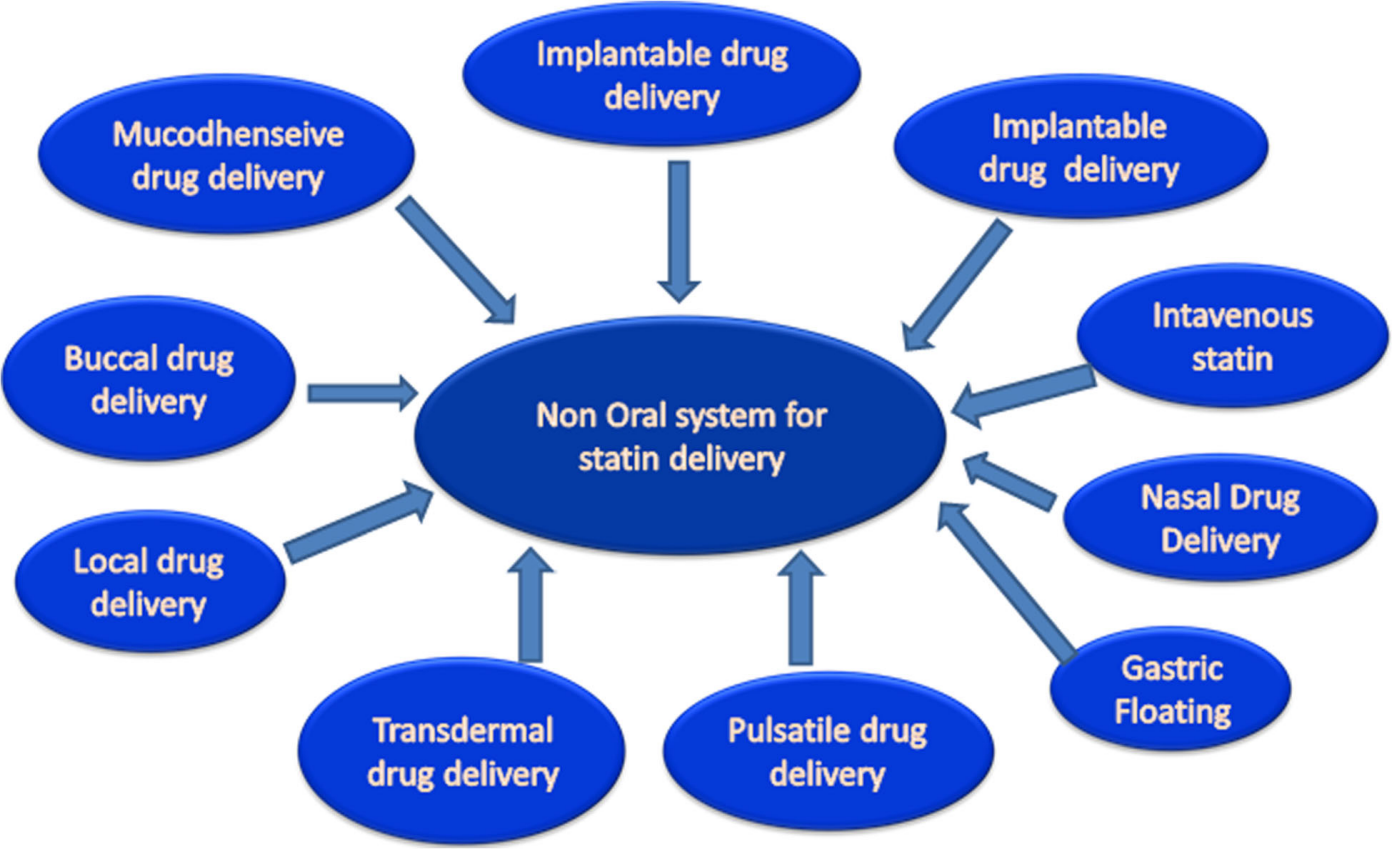
Non-oral routes for enhancing the delivery of statins

Besides challenges with limited bioavailability, which has prompted alternative routes of statin administration and novel statin formulations, statins also pose a risk, albeit a somewhat reduced risk, of adverse side effects. For example, although statins confer several beneficial lipid-independent pleiotropic actions on the body such as anti-thrombotic, antioxidant, and anti-inflammatory properties [3–9], there are some statin-treated patients that develop statin intolerance [10]. Statin intolerance generally manifests as statin-associated muscle symptoms (SAMS) with a spectrum of symptoms ranging from myalgias to life-threatening rhabdomyolysis, although, as mentioned above, the likelihood of developing rhabdomyolysis is fairly small [10]. Furthermore, there is some evidence that suggests the presence of a residual risk of developing cardiovascular disease in statin-treated patients despite these patients having met their goals with regard to the lowering of low-density lipoprotein (LDL) cholesterol. Therefore, these concerns have also motivated the search for new pharmaceutical formulations for statins and drug delivery methods/strategies for their administration, as well as non-oral routes for statin administration (Fig. 1), with the goals of increasing their overall bioavailability, therapeutic efficacy, and limiting their possible side effects. Hence, this review will evaluate non-oral routes of statin administration and novel dosage forms (formulations) for their delivery in order to improve their therapeutic effectiveness by using smaller doses and thereby limiting any potential side effects.

### Buccal drug delivery

Orabase was introduced for the first time in 1947 as a buccal drug delivery system. The natural gum tragacanth was combined with a dental powder to provide penicillin to the oral mucosa [11]. Mucoadhesive buccal drug delivery systems are particularly useful for drug administration when the incorporated drug is prone to significant degradation in gastrointestinal fluids, subject to extensive first-pass metabolism, possess short half-lives of elimination, require continuous and controlled delivery, and have low aqueous solubility. All of these features contribute to limit the bioavailability of a drug if given by oral administration in which the patient swallows the drug product together with some form of liquid to assist with the swallowing process. Drug administration can be accomplished using the buccal mucosa because of the relatively smooth and constant surface of the buccal mucosa and its rich vascularity [12]. Various dosage forms have been developed for buccal drug delivery including adhesive tablets, gels, patches, and ointments. Typically bioadhesion of the dosage form to the underlying buccal mucosa is accomplished using different types of hydrophilic polymers. Lovastatin (LOV) is an example of a statin drug that has very low and variable bioavailability, because it undergoes extensive first-pass metabolism in the liver. A LOV-containing mucoadhesive buccal tablet has been, which greatly improved the overall bioavailability of LOV [13].

In another separate study, pravastatin sodium was incorporated into a bilayered, mucoadhesive buccal tablet with a matrix containing carrageenan gum. To improve the tablet’s overall performance, magnesium oxide was included in the formulation. Furthermore, to ensure unidirectional release of the pravastatin, the tablet was coated with ethyl cellulose, which functioned as an impermeable support layer. A diverse group of permeation enhancers were evaluated to enhance the penetration of pravastatin into the buccal mucosa. A formulation that was comprised of 1% sodium lauryl sulfate displayed excellent permeation of pravastatin through the buccal mucosa. Subsequent histopathological investigations revealed no mucosal injury from the incorporation of 1% sodium lauryl sulfate as the permeation enhancer. Therefore, buccal transmucosal delivery of pravastatin is a suitable replacement for the more traditional oral route of pravastatin administration [14]. In yet another study, Asha et al. formulated different types of mucoadhesive buccal tablets incorporating atorvastatin calcium, which also included diverse polymers such as Carbopol 934P (CP), hydroxyethyl cellulose (HEC), sodium carboxymethyl cellulose (Na-CMC), ethyl cellulose, and sodium alginate. It was determined that mucoadhesive buccal tablets containing a 3:2 ratio of CP and Na-CMC showed the greatest percent of drug release and that there was no decomposition of atorvastatin during the 6-h in vitro release study. Therefore, these results would suggest that the mucoadhesive buccal tablets containing atorvastatin represent a drug delivery strategy to bypass hepatic first-pass metabolism and increase overall bioavailability [14].

### Mucoadhesive drug delivery systems

Mucoadhesive drug delivery systems achieve mucoadhesion by the judicious selection and subsequent incorporation of natural and synthetic polymers. During hydration, these polymers become adhesive to a mucous membrane and facilitate protracted drug delivery at the site of their application. This drug delivery platform continues to be of interest to pharmaceutical scientists, because it represents a method of drug delivery that avoids drug degradation and inactivation in the gastrointestinal environment and also as a means to bypass the first-pass effect that results when drug is delivered to the liver from the portal vein after drug absorption in the gastrointestinal tract [15].

Microcapsules of simvastatin (SIM) have also been prepared in combination with hydroxypropyl beta-cyclodextrin (HPBCD) in an attempt to achieve more efficient drug delivery. The use of HPBCD allowed for greater drug loading, as well as an extended duration of drug release (12 h). Furthermore, in this investigation, it was demonstrated that extracts from *Dillenia indica* (‘elephant apple’ evergreen tree native to East Asia) could be utilized to impart adequate mucoadhesive properties to the microcapsules. Lastly, it was shown that SIM had adequately interacted with HPBCD, which led to an increase in aqueous solubility and a greater rate and extent of drug release as observed with in vitro dissolution profiles. The in vivo findings demonstrated increased bioavailability and an enhanced antihyperlipidemic effect for 24 h due to the controlled release of SIM [16].

In another study, microspheres containing SIM were prepared using polymers such as hydroxypropylmethyl cellulose (K 100 M), Na-CMC, CP, sodium alginate, guar gum, ethyl cellulose, xanthan gum, methyl cellulose, and a solution of 10% calcium chloride. Subsequent in vitro experiments demonstrated that the formulation designated as F10, which utilized a mixture of alginate and methyl cellulose, possessed significant mucoadhesive properties. The rate and extent of SIM release from the microcapsules in vitro depended on the composition of the microcapsule’s coating. The formulation designated as F10 showed protracted release of SIM in vitro over 8 h [17].

### Gastric floating

Floating drug delivery systems (FDDS) are buoyant in the gastric fluids of the stomach due to their low density and are able to remain in the stomach for an extended period of time without negatively impacting the normal gastric emptying time to any significant degree. Rapid transit through the gastrointestinal tract limits the amount of drug that can be released from a conventional dosage form and, therefore, the overall bioavailability, since the majority of drug absorbed after oral administration is from the stomach and especially the duodenum of the small intestine. FDDS systems overcome this limitation by increasing the retention time or residence time in the stomach, which allows extra time for drug release, dissolution (especially for drugs that are slightly less soluble in the gastric pH compared to the higher pH of the duodenum), and absorption to occur and increase overall bioavailability [18].

Pravastatin FDDS have been prepared using diverse viscosity grades of hydroxypropyl methylcellulose (HPMC) (HPMC K4M, HPMC K100 M, and HPMC K15 M) and with varying the ratio of drug to polymer. The pravastatin FDDS showed a significant increase in absorption and improved bioavailability due to increased residence time of pravastatin in the stomach [19].

### Colon drug delivery systems (CDDS)

Targeting statin delivery to the colon has been attempted in the past. Formulations of statins for absorption in the lower gastrointestinal tract and the colon are designed for once a day dosing and rapid release upon their arrival in the lower gastrointestinal tract. CDDS formulations provide a remarkable increase in the plasma concentration of statins and the ability to maintain the plasma concentration of the statin for 12 to 24 h following the initial ‘burst’ of drug release once at the site of delivery. Moreover, this dosage form leads to the production of active metabolites of some statins most likely through either the effects of the natural flora in the colon, or via other metabolic pathways, and leads to better clinical results in terms of plasma lipid lowering. These benefits are not usually attained with either systemic, or conventional oral delivery of statins [2].

### Pulsatile drug delivery systems

Pulsatile drug delivery systems are most appropriate for diseases in which drug release is designed to correspond to the rhythms of the illness in an effort to optimize therapy while simultaneously minimizing a drug’s potential adverse side effects. For example, the synthesis of cholesterol by the liver is greater during the night than it is during the daytime. Daytime cholesterol synthesis accounts for approximately 30–40% of diurnal cholesterol synthesis, although the highest production occurs in the morning [20]. Therefore, there is a need for novel drug delivery systems that can supply drug at precise levels to coincide with the daily rhythms of physiological processes in the body. These dosage forms would appear to have various benefits, including a more steady delivery of drug during the precise time of the day that the pathological process is most severe, a reduction in the total amount of drug needed for the dosage form, a decrease in the frequency of drug dosing/administration, a reduction in adverse drug reactions, and enhanced patient compliance.

Several statin formulations are commonly designed for once daily dosing in the evening to correspond with the increased synthesis of cholesterol during the evening hours, and especially during the initial morning hours when cholesterol neosynthesis is maximal. A pulsatile drug delivery system comprised of lovastatin-filled microspheres has been prepared for administration just prior to sleep. This delivery system is able to release drug in the early morning hours when cholesterol biosynthesis reaches a maximum. The delayed time of release is 5 h and the total duration of lovastatin release is 12 h, which is compatible with so-called ‘chrono-modulated therapy’ of a statin for hepatic cholesterol synthesis [21].

### Local drug delivery

Recent investigations have shown that many problems in periodontal disease can be positively correlated to different microorganisms, especially Gram-negative bacteria. Periodontal disease usually results in chronic inflammation in the oral cavity and oftentimes mediates bone resorption and creates defects in underlying bone. The goal of current periodontal therapy is to suppress or eliminate the pathogenic bacteria from the tooth surface and to alter this microbiota to one that is non-pathogenic for the teeth. Systemic antimicrobial agents have traditionally been used for severe periodontal infections. However, this therapeutic approach can lead to bacterial resistance, drug toxicity, and drug interactions that limit the use of this treatment strategy. Fortunately, local drug delivery has the potential to limit the side effects that are often associated with systemic antimicrobial therapy, but a limitation is the availability of the drug to the local site at a sufficient therapeutic concentration [22]. Many studies have shown that the local administration of simvastatin has been effective with regard to the formation of bone. Therefore, local delivery of simvastatin in the oral cavity, specifically in the periodontal pocket, has been suggested as regenerative therapy for bone loss secondary to chronic periodontitis [23].

### Intravenous statin formulations

Intravenous drug delivery is frequently employed due to a rapid onset of the desired pharmacological effect. Prinz et al. demonstrated that an intravenous formulation of rosuvastatin, when given four hours after ischemia and at a dose of ~ 0.2 mg/kg, protects focal brain ischemia/reperfusion in the rat. In fact, the stroke-protective influence of intravenous rosuvastatin was maintained for five days after ischemia was experimentally-induced [24]. Intravenous rosuvastatin offered neuroprotection at rosuvastatin plasma levels less than 0.5 ng/ml and was suggested to be related to elevated concentrations of phosphorylated eNOS and phosphorylated Akt kinase in the vasculature. Therefore, these finding with intravenous rosuvastatin in the rat would suggest a potential therapeutic benefit for the treatment of acute stroke in humans using intravenously-administered statins [24].

### Transdermal drug delivery system (TDDS)

TDDS is a class of controlled drug delivery systems in which the route of drug delivery is through the skin with the goal of achieving a predetermined and regulated rate of drug administration [25]. This non-invasive drug delivery system has several advantages including improved patient compliance, convenience, ease of use, and reduced fluctuation in the drug plasma concentration and, consequently, a reduced likelihood of drug overdose [26]. Moreover, it avoids the harsh acidic and enzymatic environment of the gastrointestinal tract, as well as the potential for drug/food interactions and the so-called ‘first-pass’ effect of metabolism by the liver. These features prolong the drug’s pharmacological effect and also maintain drug stability by avoiding chemical- and/or enzymatic-based degradation of the drug. Translocation of drug across the stratum corneum of the skin is the rate-limiting step during the transdermal permeation of most drugs. However, continued advances in nanotechnology may offer a solution to overcome this limitation [27]. Simvastatin-loaded niosomal gels have been prepared by Zidan et al. in order to improve the hypolipidemic efficacy of this statin. The pharmacokinetic results in rats demonstrated that the transdermal niosomal formulation increased the bioavailability of simvastatin approximately 3-fold when compared to a conventional oral tablet. Therefore, this simvastatin-loaded niosomal gel formulation may represent a suitable TDDS for the controlled delivery of a statin drug that results in greater overall bioavailability and drug efficacy [28].

In another study, Xiang et al. designed an injectable subcutaneous gel formulation to extend the release of pitavastatin calcium to treat hyperlipidemia. The injectable pitavastatin gel formulation incorporated a high concentration of phospholipids and soybean oil, which were mixed with ethanol and produced a low-viscosity, phospholipid-based platform that existed in a ‘sol’ state. After subcutaneous injection, the formulation gelled and resulted in the formation of a subcutaneous drug reservoir of pitavastatin in vivo. In vitro drug release studies demonstrated that the pitavastatin gel formulation released pitavastatin for 15 days. Thus, this particular formulation demonstrates the feasibility of delivering a statin drug (pitavastatin) to potentially treat hyperlipidemia for an extended period of time post-dosing [29].

### Nasal and inhalation drug delivery targeting the lung

In asthma, the airway epithelium is the primary player in terms of remodelling and airway inflammation [30]. During inhalation, a drug is delivered directly into the airway without extensive clearance by first-pass metabolism in the liver, thereby maximizing the distribution of the drug to the target site. Therefore, direct administration of statins to the lungs via inhalation may be a strategy to circumvent first pass metabolism normally observed after oral administration and may allow for smaller doses of statins to be administered. Importantly, smaller doses of a statin may prevent ‘off-target’ side effects related to the oral ingestion of statins, for instance, myopathy, which is the most common adverse side effect [31]. Presumably, inhaled statins would potentially lead to a decrease in toxicity due to more efficient delivery of the drug to the systemic circulation at a reduced dose [30].

Previous studies have shown that statins may also produce anti-inflammatory, anti-oxidant, and immunomodulatory effects, which make them suitable drug candidates for airway inflammatory disease. Xu et al. demonstrated that treatment with simvastatin via either inhalation (1, 5, 20 mg/mL), or intratracheal (i.t.) instillation (2 mg/kg), in an avalbumin mouse model of allergic asthma led to a noticeable inhibition of airway inflammation, airway mucus production, and lung eosinophilia. Surprisingly, simvastatin’s anti-inflammatory effect was similar to that observed with dexamethasone (1 mg/kg) administered by intraperitoneal injection [32]. In yet another study, Tschernig et al. showed that ultra-low doses of simvastatin (0.06–6 μg/kg) administered by intranasal (i.n.) inhalation prevented airway inflammation and airway resistance triggered by murine house dust mites [33].

### Implantable drug delivery systems (IDDSs)

Implantable drug delivery systems (IDDSs) are surgically implanted and function as a reservoir or depot for the protracted delivery of various drugs [34]. Thylin et al. showed that simvastatin- loaded implants (120 mg/kg of body weight) improved bone formation and increased the rate of bone growth in a murine calvarial model [35]. In another study, a simvastatin–PLGA scaffold was designed as a carrier for simvastatin and implanted into the cavities of mandibular incisors of Wistar rats to achieve bone replacement/regeneration. The findings showed that local application of simvastatin effectively supported alveolar bone by facilitating the formation of bone within the cavities. In still another study, a local statin implant was prepared by dissolving 200 mg of polylactic (MW = 20,000 D), with or without 50 mg of simvastatin, in 2 mL of acetone and injected into a round glass mold (15 mm diameter, 2 mm depth) in an effort to repair and restore a bone defect. The underlying mechanism suggested for restoration of the bone defect by simvastatin was the increased expression of hypoxia-inducible factor-1alpha and BMP-2; thus, enhancing the angiogenic and autogenous osteogenic potential of stem cells in the defective bone region [36].

## Conclusion

Statin administration by the oral route of drug delivery is not always optimal in terms of absorption due to limitations in overall bioavailability. Therefore, new drug delivery strategies have been designed to improve the bioavailability, solubility, and effectiveness of statins (Tables 1 and 2). Mucoadhesive buccal drug delivery systems are used for drugs that are degraded in the gastrointestinal tract and undergo extensive first pass metabolism when administered orally in an attempt to increase their bioavailability. Gastric floating drug delivery systems improve the solubility of certain drugs that may be less soluble at the higher pH normally found in the upper portion of the gastrointestinal tract (duodenum) by creating a longer time for dissolution to occur in the more acidic environment of the stomach. Pulsatile drug delivery systems are dosage forms/formulations that respond to the normal circadian rhythms of the body. For example, this type of drug delivery system can be highly efficacious in lowering cholesterol, since the biosynthesis of cholesterol follows a normal circadian rhythm where production is greatest during the night and maximal in the very early morning hours. Local oral drug delivery suggests another alternative method for drug administration. This approach has several advantages, such as rapid access to the bloodstream, non-invasiveness, suitability for topical treatment, no requirement for larger doses of the drug due to efficient absorption, and a reduction in systemic toxicity. The intravenous route of drug administration makes possible the rapid attainment of blood concentrations, and, consequently, the desired pharmacological response, so that the administered drug is clinically effective. The transdermal route of drug delivery has different advantages when compared to the traditional oral route of drug administration. Advantages of transdermal drug delivery include effective absorption of lipophilic drugs, prevention of first-pass metabolism, release of the incorporated drug for longer periods of time, decreased side effects, and the capacity to maintain a fairly constant blood level of the drug over time. The nasal route of drug delivery for subsequent inhalation to and absorption from the lungs has certain advantages when compared to other non-invasive routes of drug delivery, which includes, as an example, very rapid absorption due to the extremely large surface area of endothelium available for drug absorption in the lungs, as well as rapid absorption of drugs across the nasal mucosa following nasal instillation. In comparison to the intranasal route of drug administration, whether that be for drug delivery to the brain or delivery to the lungs following inhalation, IDDSs have the advantage of being implanted directly at the target site to release drug locally and at a controlled rate for an extended period of time. CDDSs allow drug delivery not only locally within the large intestine, but also provide for the systemic absorption of drugs. As mentioned in this review, the colon has less hostile environment than the small intestine and stomach in terms of drug decomposition/degradation mediated by pH or enzymes. While this route of drug administration/delivery typically results in less drug being absorbed into the systemic circulation, nevertheless, drugs may experience a longer retention time in the large intestine, which allows for greater drug release and, consequently, increased local drug concentrations.

In conclusion, due to several limitations associated with the oral administration of statins, which include limited overall bioavailability, first-pass metabolism, less than optimal aqueous-solubility, and systemic side effects, non-oral delivery systems for the administration of statins have been investigated for this important and fist-line class of hypolipidemic agents.

**Table 1:** Examples of parenteral statin delivery systems

System	Drug	Polymer	Dosage form	Plasma level	reference
Buccal drug delivery	Lovastatin	Carbapol 934P, PVP K30, HPMC K4M, HPMC K100 M	Buccoadhesive pill	NM	[13]
Buccal drug delivery	Pravastatin sodium	Carrageenan gum	Bilayered buccal pills	NM	[37]
Mucoadhesive drug delivery	Simvastatin	Hydroxy propyl beta cyclodextrin (HPBCD)	Microcapsules	NM	[16]
Mucoadhesive drug delivery	Simvastatin	Sod.Alginate: Methyl cellulose (F10)	Microspheres	NM	[17]
Gastric floating	Pravastatin	Hpmc k4 m, hpmc k15 m, and hpmc k100 m.	Floating pills	NM	[19]
Pulsatile drug Delivery	Lovastatin	Karaya gum/kondagogu Gum/xanthum gum/guar gum	Microspheres	NM	[21]
Local drug delivery	Simvastatin	–	Local delivery	NM	[23]
Intravenous drug delivery	Rosuvastatin	–	Rosuvastatin was dissolved in normal saline and administered intravenously	0.5 ng/ml (with the 0.2 mg/kg dose)	[24]
Transdermal drug delivery	Simvastatin	Sorbitan monolaurate and sorbitan monostearate (span 20 and 60	Niosomal gel	NM	[28]
Transdermal drug delivery	Pitavastatin	Phospholipids and soybean oil	Gel	301 ng/mL 8 h 10 ng/mL for at least 15 days	[29]
Nasal drug delivery	Simvastatin	–	Jet nebulizer	0.260 μg/ml during 6 h(with the 5 mg/ml dose, 10 min)	[32]
Nasal drug delivery	Simvastatin	–	Intranasal	NM	[33]
Implantable drug delivery systems	Simvastatin	Polylactic acid	Gel	NM	[35]
Implantable drug delivery systems	Simvastatin	Polylactic acid/polyglycolic acid	Implant	NM	[36]

*NM* not mentioned

**Table 2:** Advantages and disadvantages of different parenteral statin delivery systems

Delivery system	Advantages	Disadvantages	Reference
Buccal drug delivery	■Bypasses the hepatic first-pass ■Bypassing the hepatic first-pass metabolism ■ high bioavailability ■ patient compliance improvement ■facility of availability of absorption site ■Sustainment of drug delivery ■ simple drug administration ■appropriate for drugs irritating mucosa mildly and reversibly ■ pain-free administration ■simplicity of drug withdrawal ■improvement of drug formulation by adding a pH modifier, enzyme inhibitor or permeation enhancer	■restricted the area of absorption ■postponing the extent and rate of drug absorption via the mucosa by obstacles like mucus, saliva, basement membrane, and membrane covering granules, ■drug dilution by constant saliva secretion (0.5–2 L/day) ■ Choking risk by non-voluntarily swallowing ■elimination of dosage form by non-voluntary swallowing of saliva leading to waste of dissolved drug .	[38, 39]
Mucoadhesive drug delivery	■ localized and targeted dosage form at a particular area ■ increasing drug flux at the targeted tissue ■ high bioavailability ■ bypassing first pass metabolism and low enzyme activity ■ sustainment of drug delivery ■ pain-free administration	■ incidence of local ulcers because of extended contact of the dosage form ■ lack of ability to recognize proper drugs for this delivery system due to absence of suitable model for screening ■poor patient tolerability regarding irritancy and taste ■ forbidden drinking and eating	[40]
Gastric floating	■ drug absorption improvment ■controlled drug delivery ■minimizing the mucosal irritation ■ suitable for treatment of gastrointestinal disorders ■simple and typical facilities for producing site-specific dosage forms ■simple drug administration ■ patient compliance improvement	■ not suitable for drugs with low solubility or stability or in stomach ■ requiring great amount of water (200–250 ml) in the stomach ■ not convenient candidates for drugs injuring gastric mucosa ■ not suitable candidates for drugs absorbing across the whole GIT and undergoing first pass metabolism like nifedipine	[41]
Pulsatile drug Delivery	prolonged day or night time activity ■reducing side effects by decreasing frequency and size of dosage form ■improvement of patient compliance. ■ suitable for circadian rhythms of disease or body activities ■ targeted drug delivery to a particular area like colon ■ protecting mucosa from irritating drugs ■ bypassing first pass metabolism ■ providing steady drug dosage at the targeted area ■ preventing the peak-valley fluctuations	■ low capacity of drug loading ■imperfect drug release ■numerous steps for drug production ■ more expensive production	[42]
Local drug delivery	■ elevated concentration in subgingival area ■independent of patient compliance ■ not harmful for the significant advantageous microflora of GI tract ■ bypassing systemic intolerance	■hardness in locating of dosage forms of the antimicrobial agents in deeper areas ■placing should be professionally ■patient compliance is needed for placing manually ■incomplete drug penetration	[43]
Intravenous drug delivery	■ possibility of self-administration of drug in controlled/constant manner, undesirable effects of drug administration can be ceased by removing patch. ■ not affecting drug delivery by food and gastrointestinal disorders (diarrhea or vomiting) ■ bypassing first-pass metabolism in the liver, decreasing the level of dosage form, and therefore reducing side effects of drug. ■suitability of administration for patients with facial injuries ■ decreasing frequency of drug dose ■non-invasive administration and improving patient compliance ■simplicity of production and transportation	■difficulty in Large dose administration ■ not proper for drugs with size 500 Da ■ difficulty in obtaining high plasma level of ■ possibility of allergic or irritating reactions using high drug dosage form ■ variability in skin permeability from one area to another in same person and also in one person to another ■difficulty in contact between device and skin, because of wetting skin during bathing and sweating, leading to device falloff	[44]
Transdermal drug delivery	■extended time of drug function ■decreasing the frequency of dosage form ■More steady plasma level of drug ■Improving bioavailability ■Reduction of adverse effects ■Flexibility of withdrawal drug administration by easily taking the patch from the skin	■probability of local irritation at the area of administration ■ probability of skin irritation or contact dermatitis because of drug or excipients ■limited number of delivered drugs due to low permeability of skin	[45]
Nasal drug delivery	■ lack of drug degradation in GI tract ■bypassing hepatic first pass metabolism ■fast drug absorption ■ improving bioavailability of bigger drug molecules using absorption enhancer or other methods ■great nasal bioavailability for smaller drug molecules ■possibility of drug delivery to the systemic circulation via nasal delivery for drugs not absorbing orally ■a suitable alternate to parenteral path, particularly, for peptide and protein drugs. ■appropriate for the patients, particularly for ones on long term therapy, in comparison with parenteral path ■great drug dose absorption due to large nasal mucosal surface site ■quick drug absorption through highly-vascularized mucosa ■quick beginning of function ■simple and non-invasive drug administration ■bypassing the first-pass metabolism ■improvement of bioavailability ■lower required dose therefore, reducing drug side effects ■minimal aftertaste ■ improvement of patient compliance ■self-administration	■ a smaller absorption surface area compared to gastrointestinal tract ■ more possibility of irritation compared to the oral delivery system. ■ possibility of occurring local side effects and irreversible cilia injury on the nasal mucosa due to added substances to the drug ■ possibility of a mechanical loss of the drug within the other regions of the respiratory tract such as lungs because of the unsuitable administration procedure ■possibility of disruption and even dissolution of membrane due to applied certain surfactants as chemical enhancers	[30, 46, 47]
Implantable drug delivery systems	■localized delivery ■ patient compliance improvement ■ lower required dose therefore, reducing drug side effects ■ drug stability improvement ■suitable for direct administration ■ simplicity of drug withdrawal	■higher intricacy ■more expensive ■lack of accessibility of polymers ■requirement to certain physical characteristic like mechanical strength and adjustable degradation kinetics	[48]

## Data Availability

This is a review article and there is no raw data.

## Abbreviations

CDDS: Colon drug delivery systems
CP: Carbopol
FDDS: Floating drug delivery systems
HEC: Hydroxyethyl cellulose
HMG-CoA: 3-hydroxy-3-methyglutaryl coenzyme A
HPMC: Hydroxypropyl methylcellulose
HPβCD: Hydroxypropyl beta-cyclodextrin
IDDS: Implantable drug delivery system
LDL: Low-density lipoprotein
LOV: Lovastatin
Na-CMC: Sodium carboxymethyl cellulose
SAMS: Statin-associated muscle symptoms
SIM: Simvastatin
TDDS: Transdermal drug delivery system
